# Screening for urinary biomarkers of steroid-resistant nephrotic syndrome in children

**DOI:** 10.3892/etm.2012.875

**Published:** 2012-12-21

**Authors:** YONGQI BAI, WENJUN LIU, QULIAN GUO, YAN ZOU

**Affiliations:** Department of Pediatrics, Affiliated Hospital of Luzhou Medical College, Luzhou, Sichuan 646000, P.R. China

**Keywords:** urinary proteomics, urine, steroid-resistant, biomarkers, nephrotic syndrome

## Abstract

The present study aimed to screen for urinary biomarkers of steroid-resistant nephrotic syndrome (SRNS) in children. These biomarkers were divided into three groups, the control, the steroid-sensitive nephrotic syndrome (SSNS) and the SRNS groups, which were composed of 45, 32 and 9 children, respectively. Urine samples were obtained and analyzed using Au-chips. Compared with the control group, the peak intensities of four proteins, measured using mass-to-charge ratios, were significantly increased in the primary nephrotic syndrome (PNS; SSNS and SRNS combined) group (P<0.01). The intensity of three and one peaks increased significantly in the SSNS and SRNS groups, respectively, compared with the control (P<0.01). Compared with the SRNS group, the intensity of one protein peak increased in the SSNS group (P<0.01). The diagnostic model was established based on these four protein peaks. The sensitivity and specificity of the model were 88.89 and 93.75%, respectively. Four differentially expressed proteins may consequently serve as urinary biomarkers for SRNS in children.

## Introduction

Primary nephrotic syndrome (PNS) is one of the most common kidney diseases among children. Glucocorticoids (GCs) are drugs frequently used in the treatment of PNS. Although GCs greatly reduce the mortality rate in PNS patients, 60 to 80% of steroid-responsive patients suffer from proteinuria relapse, steroid-dependent nephritic syndrome and even steroid-resistant nephrotic syndrome (SRNS) following complete remission at the early stage of hormonal therapy (1). In total, >10% of SRNS cases develop into end-stage renal diseases due to the lack of a treatment protocol at the early stage (2). Therefore, steroid resistance has become the most difficult problem to overcome in PNS treatment. As a reaction of the body to drugs, steroid resistance, whether caused by abnormal receptor genes (3), disproportion in the receptor protein structure (4) or protein phosphorylation of the post-receptor transduction pathway, is realized by changing the protein levels in PNS patients. Scholars have demonstrated that certain genetic mutations (5,6) and membranous nephropathy caused by viral hepatitis type B (7) are closely correlated with SRNS. The protein expression levels of multidrug resistance-1 (MDR-1) and P-glycoprotein 170 (Pgp170) serve as predictors for hormonal responses (8,9). However, none of the mentioned studies were able to make a complete and systemic judgment on the varying responses of hormone therapy in PNS patients. Thus, these studies all have a low specificity. Presently, there is neither a diagnostic test which precisely detects SRNS (10) nor a protocol design for an early-stage treatment of SRNS.

With the development of proteomics, the application of urinary proteomics has become more and more extensively explored for the treatment of kidney diseases (11,12), particularly in newborns and children (13). Based on urinary proteomics, the present study aimed to seek the urinary biomarkers for the early diagnosis of SRNS in children.

## Subjects and methods

### Subjects

Patients involved in the present study received treatment at the Affiliated Hospital of Luzhou Medical College between September 2009 and December 2010. These patients were divided into two groups, the SRNS and steroid-sensitive nephrotic syndrome (SSNS) groups. The SRNS group consisted of 9 children with an average age of 5.4±3.1 years, of whom 6 were boys and 3 were girls. They all met the basic diagnostic criteria for SRNS (1), namely that urinary protein remained positive subsequent to eight weeks of prednisone treatment. The SSNS group consisted of 32 children with an average age of 5.0±3.8 years, of whom 20 were boys and 12 were girls. They also all met the basic diagnostic criteria for SSNS (7), namely that urine protein was negative following glucocorticoid treatment (prednisone 1.52 mg/kg daily) for <8 weeks, and that the negative result remained following the decrease in hormone levels. The control group consisted of 45 healthy children with an average age of 5.1±3.5 years, of whom 30 were boys and 15 were girls. There were no statistical differences in age between the three groups (P>0.05). The study was approved by the ethics committee of the Affiliated Hospital of Luzhou Medical College, Luzhou, Sichuan, China. Written informed consent was obtained from the patient’s family.

### Preparation of urine samples

Urine samples of patients who met the conditions were collected within 24 h of each other. A total of 20 ml of urine was obtained, kept in the refrigerator at 4°C for half an hour and centrifuged at 3,000 rpm at 4°C for 5 min. The supernatant liquid was subpackaged into 0.5-ml tubes containing 10 to 100 *μ*l each and then kept at −80°C. A minimum of three tubes were prepared for every sample. All procedures were performed below 4°C, and all samples were frozen and thawed only once. Ten portions consisting of 2 ml urine were obtained from the control group and centrifuged, then kept at −80°C. Protease inhibitors were added to the collected specimens.

### Protein chip detection

Given that the protein content in urine is markedly lower than that in blood, blood chips were not suitable for use in the present study. Au-chips (ChipHergen, Fremont, CA, USA), a type of protein chips with Au-plated ponds, were used for protein selection in this study. Au-chips do not capture proteins; these chips bear them. In addition, they are able to reflect the expression of all proteins.

### Preparation of half-saturated erucic acid solution

A solution (200 *μ*l) containing 50% methyl cyanides and 0.5% trifluoroacetic acid was prepared. A total of 100 *μ*l of the prepared solution was added to overdosed erucic acid powder, oscillated and then centrifuged at 10,000 rpm for 3 min. The supernatant liquid was then withdrawn and double-diluted with the prepared solution. The mixture was kept in the dark and was used on the same day.

### Sample application

The urine samples were removed from the freezer, thawed on ice for 30 to 60 min and centrifuged at 10,000 rpm at 4°C for 2 min. Exactly 10 *μ*l of the supernatant liquid and 10 *μ*l of the half-saturated erucic acid solution were added into the centrifuge tube (0.2 ml). The prepared solution was mixed thoroughly. The mixture was then applied into the Au-chip ponds, with 1 to 2 *μ*l of the mixture in each chip, and then dried. According to the protein content in the sample, another 1 *μ*l of the half-saturated erucic acid may have been added to the pond if necessary. A single pond on each different chip was randomly selected for the application of the control urine to evaluate the variability of the different chips. Control urine was subjected to the same procedures.

### Chip detection

Chips were processed using a Protein Biological System II mass spectrometer at a laser intensity of 210, detector sensitivity of 9 and optimized range of 2,000 to 20,000 Da. Simulated spectra were generated by the computer.

### Statistical analysis

Biomarker Wizard 3.1 software was adopted for cluster and other analyses. The results from the ponds of control urine showed that the coefficient of variability among the different chips was <10%. The threshold of frequency for the significant protein peak was set to 10%. Signal to noise (S/N) ratio filtration was performed twice. For the preliminarily screened protein peak, a t-test was carried out which involved a comparison between the three groups. P<0.01 was considered to indicate a statistically significant difference.

### Establishment of the diagnostic model

Distinctive protein mass-to-charge ratio (m/e) peak values were considered as the input layer and peak height as the quantitative index. The expected output values of SRNS and SSNS were set to 1 and 0, respectively. A diagnostic model was established using Biomarker pattern software 5.0.2. Its sensitivity, specificity and predictive value were analyzed.

## Results

### Screening of urinary PNS markers

**Figure 1.**
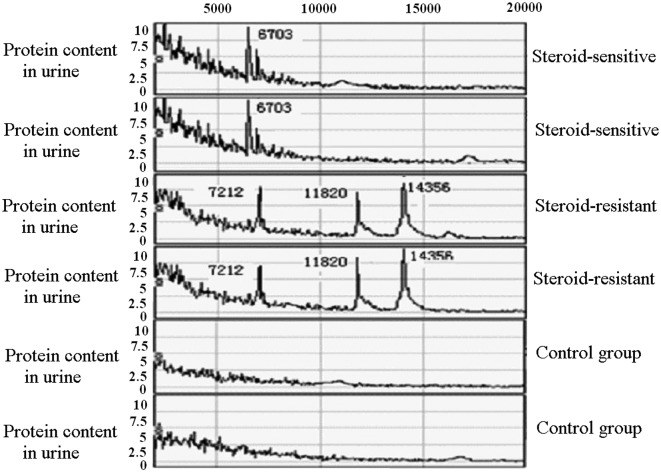
Comparisons of the spectra of differentially expressed proteins in urine between the primary nephrotic syndrome (PNS) and control groups.

**Figure 2.**
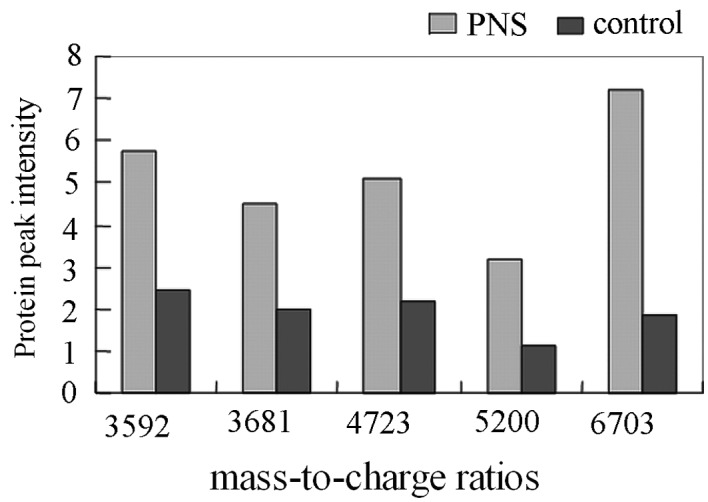
Comparison of the protein peaks between the PNS and control groups. PNS, primary nephrotic syndrome.

In every urine sample, ∼50 protein peaks were detected at the 2,000 to 20,000 range of relative molecular mass. Comparison of the mass spectra of the 41 PNS and 45 healthy children showed that there were significant differences in the 5 protein peaks. The m/e peak values were 3592.23, 3681.17, 4723.76, 5200.19 and 6703.72 (P<0.01; Table I and Figs. 1 and 2). The peak intensities of these proteins increased notably in the PNS group, which suggested that they may be protein markers for PNS. Proteins at m/e peak values of 2770.31 and 4729.15 were common proteins shared by the two groups, and were used as the internal standards in the present study.

### Screening of SRNS biomarkers

**Figure 3.**
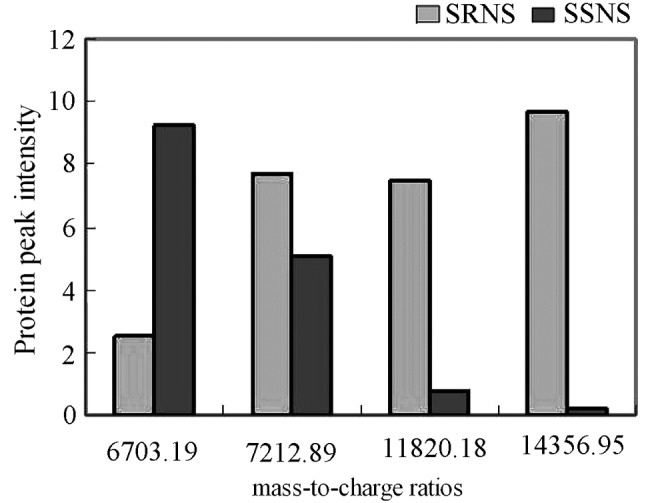
Comparison of the protein peaks between the SRNS and SSNS groups. SSNS, steroid-sensitive nephrotic syndrome; SRNS, steroid-resistant nephrotic syndrome.

The mass spectra of the SRNS and SSNS groups were analyzed and compared. Results showed that there were significant differences in the expression of four proteins (P<0.01; Table II and Figs. 1 and 3). Proteins at m/e peak values of 7212.89, 11820.18 and 14356.95 were overexpressed in the SRNS group, showing a statistically significant difference compared with the SSNS group (P<0.01). The protein at the m/e peak value of 6703.19 was overexpressed in the SSNS group, showing a statistical significance compared with the SRNS group (P<0.01).

### Evaluation of differentially expressed proteins in SRNS diagnosis

In the present study, the differentially expressed proteins in the SRNS urine were named R6703, R7210, R11820 and R14356. These proteins were used for the establishment of the SRNS diagnostic model. The protein peak heights from the mass spectra of 41 PNS patients were used as inputs for the BP neural network (an artificial neural network based on error back-propagation algorithm). The expected output values of SRNS and SSNS were then set to 1 and 0, respectively. These values were used as the cut-off point, above which was SRNS and below which was SSNS. The results showed that one case of diagnostic error was detected in the SRNS samples (1/9) and 2 cases were detected in the SSNS samples (2/32). The sensitivity and specificity of the SRNS diagnosis (Table III) were 88.89 and 93.75%, respectively.

### Protein identification

The m/e ratios of the differentially expressed proteins were used as inputs into the protein databases (http://us.expasy.org/tools/tagident.html), and their corresponding proteins were obtained (Table IV).

## Discussion

Urine is an important source of biomarkers (14), as it contains thousands of polypeptides and proteins. Changes in the structures of certain polypeptides and proteins are associated with their functions. These associations reflect the development of diseases, which not only serve as specific biomarkers for the early diagnosis and evaluation of kidney diseases, disease progression and therapeutic effects (15–17), but also for the diagnosis and follow-up of certain systemic diseases, including pre-eclampsia (18) and bladder carcinoma (19). Presently, the definitive urinary biomarkers for kidney diseases include albumin and β2-microglobulin. In the present study, a comparison of the spectra between the PNS (n=41) and control groups was performed. The result showed that proteins at m/e peak values of 3592.23, 681.17, 4723.76, 5200.19 and 6703.72 presented significant differences. These protein peak intensities increased notably in the PNS group (P<0.01), thus suggesting that they may be the biomarkers for PNS.

However, although some biomarkers for PNS have been identified, these failed to further differentiate SRNS from other PNS diseases. In the present study, a comparison between the SRNS and SSNS groups was also performed. The results showed that the protein at peak intensities of 7212.89, 11820.18 and 14356.95 was overexpressed in the SRNS group compared with the SSNS and control groups. These proteins may originate from the blood or nephridial tissues, and appear in the urine following glomerular filtration or transurethral secretion. These proteins are presumed to be steroid resistance-related proteins produced by the body which exist prior to the application of steroids. The existence of these proteins may be caused by factors which include the PNS resistance-related pathological type or genes, abnormal activities of GC metabolism-related isoforms of 11-hydroxysteroid dehydrogenase, abnormality in GC receptors and abnormality in transcription factors. Furthermore, the protein at the m/e peak value of 6703.19 was overexpressed in the SSNS group but underexpressed in the SRNS and control groups. This protein is also presumed to be a steroid sensitivity-related protein produced by the body.

Urine has an important specificity and sensitivity in predicting the development and prognosis of diseases. In addition, it may provide a reference for the early stages of treatment response. In the present study, an SRNS diagnostic model was established. The results showed that its sensitivity and specificity were 88.89 and 93.75%, respectively, in which one diagnostic error was detected in the SRNS group (1/9) and 2 errors in the SSNS group (2/32). The sensitivity and specificity of the urine proteomic diagnosis for diabetics are 89 and 91%, respectively (20). These results indicate that the four proteins detected in the urine by the present study have a high sensitivity and specificity in the screening of SRNS.

At present, there is no standard criterion for SRNS diagnosis. Therefore, the identification of early, sensitive, simple and non-invasive SRNS markers is of great significance in clinical studies. With the development of proteomics, its wide application is likely to provide a solution to this puzzle and also provide a potential substitute method to invasive nephric biopsies. Based on urinary proteomics, the present study indicates that the expression of the proteins with mass-to-charge ratios of 7212, 1182 and 14356 increase in SRNS patients. These proteins may be urinary biomarkers for SRNS.

These results demonstrate that urine proteomics plays an important role in the screening and identification of the molecular markers of children with nephrotic syndrome, particularly hormone drug-resistant nephrotic syndrome. As technology improves, further drug targets may be identified through the use of urine proteomics in the future.

## Figures and Tables

**Table I.** Comparison of the m/e ratios of protein peaks between the PNS and control groups.

		m/e				
Grouping	n	3592.23	3681.17	4723.76	5200.19	6703.72
PNS	41	5.73±1.0	4.50±2.12	5.11±1.99	3.16±0.78	7.19±1.48
Control	45	2.46±0.9	1.97±0.86	2.17±0.81	1.12±0.51	1.85±0.70
T-value		16.35	7.44	9.19	14.57	22.25
P-value		<0.01	<0.01	<0.01	<0.01	<0.01

P-values are a comparison of the m/e ratios of protein peaks between the PNS and control groups. m/e, mass-to-charge; PNS, primary nephrotic syndrome.

**Table II.** Comparison of the m/e ratios of protein peaks between the SRNS and SSNS groups.

		m/e			
Grouping	n	6703.19	7212.89	11820.18	14356.95
SRNS	9	2.54±1.17	7.71±2.76	7.52±1.32	9.69±1.53
SSNS	32	9.20±1.57	5.04±1.39	0.75±0.21	0.33±0.12
T-value		11.89	4.04	28.63	35.45
P-value		<0.01	<0.01	<0.01	<0.01

Predictive values were the number of SRNS or SSNS as judged by the BP neural network. m/e, mass-to-charge; SRNS, steroid-resistant nephrotic syndrome; SSNS, steroid-sensitive nephrotic syndrome.

**Table III.** Analysis of diagnostic efficiency of the model.

Grouping	n	SRNS (predictive value >0.5)	SSNS (predictive value <0.5)
SRNS	9	8	1
SSNS	32	30	2
Total	41	38	3

SRNS, steroid-resistant nephrotic syndrome; SSNS, steroid-sensitive nephrotic syndrome.

**Table IV.** Identification of differentially expressed proteins between the SRNS and SSNS groups.

m/e	Mean SRNS	Mean SSNS	Corresponding proteins
6703.19	2.54	9.20	50S ribosomal protein L32
7012.89	7.53	5.04	S-adenosylmethionine decarboxylase α chain
11820.18	7.52	0.75	FK506-binding protein 1A
14356.95	9.69	0.23	30S ribosomal protein S11

SRNS, steroid-resistant nephrotic syndrome; SSNS, steroid-sensitive nephrotic syndrome.

## References

[1] Hogg RJ, Portman RJ, Milliner D, Lemley KV, Eddy A, Ingelfinger J (2000). Evaluation and management of proteinuria and nephrotic syndrome in children: recommendations from a pediatric nephrology panel established at the National Kidney Foundation conference on proteinuria, albuminuria, risk, assessment, detection, and elimination (PARADE). Pediatrics.

[2] Otukesh H, Otukesh S, Mojtahedzadeh M (2009). Management and outcome of steroid-resistant nephrotic syndrome in children. Iran J Kidney Dis.

[3] Charmandari E, Raji A, Kino T (2005). A novel point mutation in the ligand-binding domain (LBD) of the human glucocorticoid receptor (hGR) causing generalized glucocorticoid resistance: the importance of the C terminus of hGR LBD in conferring transactivational activity. J Clin Endocrinol Metab.

[4] Ruiz M, Lind U, Gåfvels M (2001). Characterization of two novel mutations in the glucocorticoid receptor gene in patients with primary cortisol resistance. Clin Endocrinol (Oxf).

[5] Mir S, Yavascan O, Berdeli A, Sozeri B (2012). TRPC6 gene variants in Turkish children with steroid-resistant nephrotic syndrome. Nephrol Dial Transplant.

[6] Mbarek IB, Abroug S, Omezzine A (2011). Novel mutations in steroid-resistant nephrotic syndrome diagnosed in Tunisian children. Pediatr Nephrol.

[7] Bhimma R, Coovadia HM, Adhikari M (1997). Nephrotic syndrome in South African children: changing perspectives over 20 years. Pediatr Nephrol.

[8] Wasilewska A, Zalewski G, Chyczewski L, Zoch-Zwierz W (2007). MDR-1 gene polymorphisms and clinical course of steroid-responsive nephrotic syndrome in children. Pediatr Nephrol.

[9] Jafar T, Prasad N, Agarwal V (2011). MDR-1 gene polymorphisms in steroid-responsive versus steroid-resistant nephrotic syndrome in children. Nephrol Dial Transplant.

[10] Traum AZ (2008). Urine proteomic profiling to identify biomarkers of steroid resistance in pediatric nephrotic syndrome. Expert Rev Proteomics.

[11] Kim MJ, Frankel AH, Tam FW (2011). Urine proteomics and biomarkers in renal disease. Nephron Exp Nephrol.

[12] Siew ED, Ware LB, Ikizler TA (2011). Biological markers of acute kidney injury. J Am Soc Nephrol.

[13] Albalat A, Mischak H, Mullen W (2011). Urine proteomics in clinical applications: technologies, principal considerations and clinical implementation. Prilozi.

[14] Shao C, Wang Y, Gao Y (2011). Applications of urinary proteomics in biomarker discovery. Sci China Life Sci.

[15] Mischak H, Delles C, Klein J, Schanstra JP (2010). Urinary proteomics based on capillary electrophoresis-coupled mass spectrometry in kidney disease: discovery and validation of biomarkers, and clinical application. Adv Chronic Kidney Dis.

[16] Pejcic M, Stojnev S, Stefanovic V (2010). Urinary proteomics - a tool for biomarker discovery. Ren Fail.

[17] Wu J, Chen YD, Gu W (2010). Urinary proteomics as a novel tool for biomarker discovery in kidney diseases. J Zhejiang Univ Sci B.

[18] Carty DM, Siwy J, Brennand JE (2011). Urinary proteomics for prediction of preeclampsia. Hypertension.

[19] Schiffer E, Vlahou A, Petrolekas A (2009). Prediction of muscle-invasive bladder cancer using urinary proteomics. Clin Cancer Res.

[20] Rossing K, Mischak H, Dakna M (2008). Urinary proteomics in diabetes and CKD. J Am Soc Nephrol.

